# Comparison of Sucrose and Trehalose for Protein Stabilization Using Differential Scanning Calorimetry

**DOI:** 10.1021/acs.jpcb.4c00022

**Published:** 2024-05-11

**Authors:** Olivia Jonsson, Agnes Lundell, John Rosell, Sophie You, Kajsa Ahlgren, Jan Swenson

**Affiliations:** Department of Physics, Chalmers University of Technology, Gothenburg SE-412 96, Sweden

## Abstract

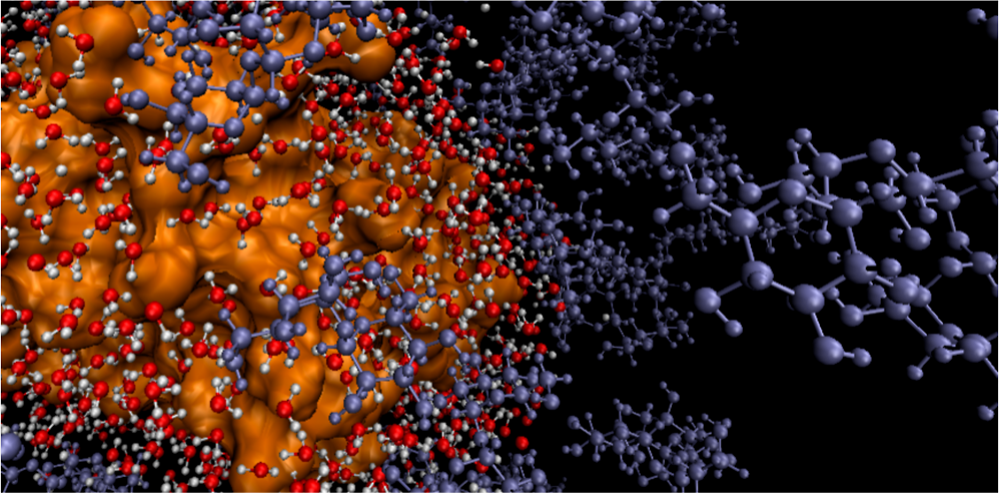

The disaccharide trehalose is generally acknowledged as a superior stabilizer of proteins and other biomolecules in aqueous environments. Despite many theories aiming to explain this, the stabilization mechanism is still far from being fully understood. This study compares the stabilizing properties of trehalose with those of the structurally similar disaccharide sucrose. The stability has been evaluated for the two proteins, lysozyme and myoglobin, at both low and high temperatures by determining the glass transition temperature, *T*_g_, and the denaturation temperature, *T*_den_. The results show that the sucrose-containing samples exhibit higher *T*_den_ than the corresponding trehalose-containing samples, particularly at low water contents. The better stabilizing effect of sucrose at high temperatures may be explained by the fact that sucrose, to a greater extent, binds directly to the protein surface compared to trehalose. Both sugars show *T*_den_ elevation with an increasing sugar-to-protein ratio, which allows for a more complete sugar shell around the protein molecules. Finally, no synergistic effects were found by combining trehalose and sucrose. Conclusively, the exact mechanism of protein stabilization may vary with the temperature, as influenced by temperature-dependent interactions between the protein, sugar, and water. This variability can make trehalose to a superior stabilizer under some conditions and sucrose under others.

## Introduction

Biological systems are often characterized by their susceptibility to changes in their environment. Changes in temperature, pH, pressure, and humidity are some of the factors that might influence the stability of a particular system.^1^ This susceptibility complicates the use of biomolecules in various applications including pharmaceuticals. Protein-based medications, such as the antibody-based cancer treatments Herceptin and Avastin, are becoming increasingly prevalent.^2^ Therefore, developing methods to enhance protein stability and reduce the sensitivity to the factors mentioned above is of great interest. A critical aspect of this is the storage of these sensitive biological and medical materials, which demands techniques that maintain their stability. Various methods have been developed for this purpose, including cryopreservation, lyophilization, and liquid formulations. What these techniques have in common is the usage of various stabilizers, where disaccharides like sucrose and trehalose have been demonstrated to be exceptional^3−5^ and even counteract denaturants.^6^ The protein-based medications mentioned above all use trehalose as a stabilizer.^2^

Cryopreservation involves cooling the material down to cryogenic temperatures, typically the temperature of liquid nitrogen (77 K),^7,8^ and is commonly used for the preservation of embryos, stem cells, and tissues. Meanwhile, lyophilization and liquid formulations are extensively used in biologics; in fact, lyophilized formulations account for 34% of all biologics and liquid formulations for 64%.^9^

One of the primary obstacles in cryopreservation is managing the crystallization of water as ice formation can severely impact biomolecules. Crystallization can force biomolecules into unfavorable conformations, leading to mechanical damage and osmotic stress, resulting in the dehydration of cells.^8,10^ Mechanical damage to cells can be caused by freezing and thawing as a result of intra- and extracellular ice formation.^11^ In fact, ice formation seems to be the largest issue with maintaining cell viability during cryopreservation.^11^ A critical factor influencing this process is the cooling rate. The cooling rate plays a pivotal role in the extent of cell damage, but both slow and rapid freezing can be problematic. A slow cooling rate tends to reduce intracellular water, since the chemical potential in the extracellular ice phase is lower than for water inside the cell.^11^ To combat these differences in chemical potential, water tends to leave the cells, resulting in dehydration and osmotic pressure.^11^ In contrast, rapid freezing limits the amount of water that exits the cells since the difference in chemical potential is not as large, leading instead to damage from intracellular ice formation.^11^ Consequently, additional methods are necessary to mitigate these challenges, such as the use of cryoprotectants.

Cryoprotectants can be categorized into two groups: permeating and nonpermeating.^7^ Commonly used permeating cryoprotectants can induce toxicity when large quantities cross the cell membrane.^12−15^ The disaccharides sucrose and trehalose are possible replacements or complements to permeating cryoprotectants.^15^ These two disaccharides possess the ability to stabilize biological materials at cryogenic temperatures, thereby preventing structural damage to biomolecules.^16,17^

The primary function of cryoprotectants is to prevent the formation of ice crystals, instead creating a glassy-like matrix that surrounds and stabilizes the molecules.^16^ The temperature at which molecular motion rapidly slows down, increasing the viscosity of a material to 10^12^ Pa s, is referred to as the glass transition temperature, *T*_g_.^18^ This temperature is commonly used as an indicator of stability, where a higher *T*_g_ indicates a higher stability as the molecules remain in a stable, glassy state at higher temperatures.^19^ However, matters might not be that simple; the glass-forming properties are not necessarily the reason behind the stabilizing function. The relation between maintaining near-native conformation in the glassy state and stability against degradation has not been found to be quantifiable and has not been established across the full range of protein–sugar compositions.^20,21^ An example of this is the polymeric carbohydrate dextran, which, despite excellent glass-forming abilities and high *T*_g_, is not able to protect lyophilized proteins as well as other cryoprotectants, e.g., disaccharides.^22^ One reason for this might be that proteins are not fully stable even below their *T*_g_, since previous studies have shown that more local relaxations (so-called secondary relaxations or β-relaxations), which are not related to the macroscopic viscosity, govern the protein stability in the glassy state.^20^

In addition to the *T*_g_, the denaturation temperature, *T*_den_, can be used to evaluate thermal stability.^23^ For both temperatures mentioned, a high temperature indicates higher stability.^19,23^ However, whether it is the same stabilizing properties of a cosolvent that give rise to a high *T*_g_ and a high *T*_den_ of the protein is still debated.^24−26^ For both sucrose and trehalose, a linear relationship between *T*_g_ and *T*_den_ has been found by Bellavia et al.^24,25^ Therefore, it was suggested^24,25^ that *T*_g_ and *T*_den_ are governed by a similar stabilization mechanism. However, it is possible that the stabilization mechanism at low temperatures, around the *T*_g_, could be different from that at higher temperatures, near the *T*_den_. For instance, at higher temperatures, hydrogen bonds are considerably weaker in relation to the thermal energy, which could alter the stabilization process. Furthermore, as found in ref (20), the stability around *T*_g_ is most likely governed by the protein dynamics, whereas the thermal stability around *T*_den_ is more determined by the thermodynamics of intermolecular interactions. However, although the exact mechanisms for protein stabilization at both low and high temperatures are somewhat unknown, different models for stabilization have been proposed. Since it has been experimentally verified that the disaccharides sucrose and trehalose are particularly successful stabilizers, especially the latter, as shown in other works,^3,27−30^ most models have been proposed to explain their stabilizing role.

In the preferential hydration model, first proposed by Arakawa and Timasheff,^31^ water molecules interact with polar parts of the protein surface, while the disaccharide does not directly bind to the protein.^32^ However, the presence of the disaccharide still stabilizes the protein through dynamic coupling between the disaccharide and the protein hydration water. Thus, by slowing down the dynamics of the hydration water, the disaccharide is able to also slow down and thereby stabilize the protein since its dynamics are “slaved” by the surrounding solvent.^33,34^ Another reason why preferential hydration enhances protein stability is due to the excluded volume effect it induces, making the native state of the protein more entropically favorable compared to its denatured state.^6,35,36^

In contrast to the preferential hydration model is the water replacement model, first proposed by Carpenter and Crowe,^37^ where the water molecules no longer bind to the protein but are instead replaced by the disaccharide, which thereby interacts directly with the protein.^32^ Therefore, the disaccharide is assumed to stabilize the protein by forming an immobile shell around it. However, neither experiments nor computer simulations have been able to support this model but rather show support for different degrees of the preferential hydration model.^31,38,39^ It is possible that the degree of preferential hydration differs between sucrose and trehalose, which might explain their varying stabilizing properties, as proposed in ref (28). Therefore, it is highly interesting to compare how sucrose and trehalose affect different thermal properties, such as *T*_g_ and *T*_den_, of proteins.

The chemical formulas of sucrose and trehalose are identical (C_12_H_22_O_11_), but sucrose is composed of the two monosaccharides, fructose and glucose, whereas trehalose is composed of two glucose molecules. This gives rise to small structural differences between the two disaccharides, which makes trehalose more likely than sucrose to form intermolecular rather than intramolecular hydrogen bonds.^32^ These structural differences likely result in differences in how these disaccharides interact with water and protein, which, in turn, should lead to different thermodynamics of the protein–sugar–water system. This can lead to differences in the protein stability. In this study, this has been investigated in detail, where differential scanning calorimetry (DSC) has been used to elucidate how trehalose and sucrose affect the thermodynamics and stability of lysozyme and myoglobin. More precisely, we have studied how the *T*_g_, the *T*_den_, and the crystallization of water depend on the content of water and sugar in relation to the protein. Through this approach, we have compared the stabilizing properties of trehalose and sucrose, gaining deeper insights into how the two disaccharides interact with the protein. Additionally, we explored the possibility of obtaining synergistic effects by mixing trehalose and sucrose. Finally, the results suggest that there is no evident relation between *T*_g_ and *T*_den_, which implies that they depend on different properties of the stabilizing disaccharide. This further implies that different types and concentrations of the disaccharide can be optimal, depending on whether it should be used for cryopreservation or to prevent protein denaturation.

## Experimental Methods

α,α-Trehalose (in dihydrated form), sucrose (in anhydrous form), lysozyme from hen egg white, and myoglobin from equine heart were all purchased from Sigma-Aldrich and used without any further purification.

### Sample Compositions

**Figure 1:**
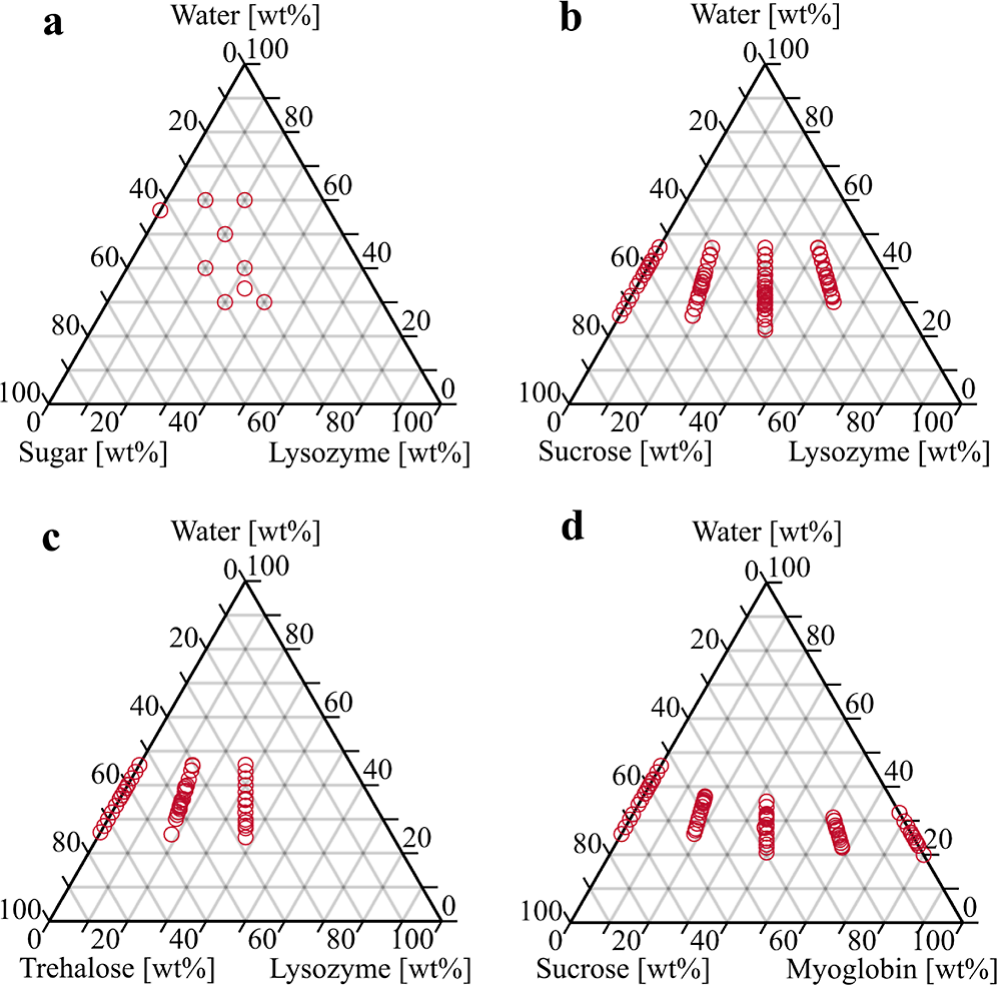
Ternary diagrams showing the measured compositions of the three-component [for (a) four-component] systems composed of (a) lysozyme/sucrose/trehalose/water, (b) lysozyme/sucrose/water, (c) lysozyme/trehalose/water, and (d) myoglobin/sucrose/water.

Each sample included varying proportions of water, protein, and sugar. The samples were prepared in three different sequences: the first two sequences used sucrose, trehalose, and lysozyme and the third sequence used sucrose and myoglobin. The difference between the first and second sequence is how the ratios between the components are varied and that the first sequence uses both sucrose and trehalose in some samples. The compositions of all samples can be seen in Figure 1. In order to explore potential synergistic effects, samples containing both single and mixed disaccharides (sucrose and trehalose) were employed in the first sequence.

The weight proportions of trehalose to sucrose were varied in increments of 25 or 50 wt %. It should be noted that for the first sequence, the phase diagram reports the total concentration of sugar, meaning that each point in Figure 1a comprises 3 or 5 data points with different compositions of the disaccharides. This means that each point in Figure 1a corresponds to two ternary systems (protein–trehalose/sucrose–water) and 1–3 quaternary systems (protein–trehalose–sucrose–water).

For the second sequence, the sugars were utilized independently of each other. These samples are seen in Figure 1b,c. The protein and sugar concentrations were varied to a greater degree, while sucrose and trehalose were not used as a mixture but separately. In this sequence, the limits for crystallization were examined more closely with a narrower concentration range of water, changing it by two percentage points. Once the limit for crystallization was approximately known, the decrements were changed to one percentage point closely around the limit. The samples for the third sequence (Figure 1d) were prepared in a manner similar to that for the second sequence, but the components used were myoglobin, sucrose, and water. For sequences two and three, the sugar to protein weight ratios were kept constant, yielding five different series: 0:1, 1:3, 1:1, 3:1, and 1:0. Depending on the solubilities of the sugar and protein in question, not all series were measured. Trehalose is less soluble in water than sucrose, while lysozyme is less soluble in water than myoglobin. Therefore, more series were prepared for the myoglobin–sucrose samples than for lysozyme–trehalose.

### Sample Preparation

The samples of the first sequence were prepared by first dissolving the sugar in Milli-Q water with stirring and heating. The protein was then added to the mixture at room temperature and stirred until it was dissolved. All samples with desired water concentrations below 35 wt % were evaporated in a vacuum chamber to reach the desired concentration.

For samples of the second sequence, the preparation method differed slightly due to the limited solubility of both sugar and protein. Two stock solutions were prepared, consisting of 50 wt % water and 50 wt % sugar (sucrose and trehalose, respectively). Lysozyme was thereafter added to the sugar solutions at room temperature and stirred until the lysozyme was dissolved. In contrast to the samples prepared with the first sequence, all samples of the second sequence contained excess water, and the desired weight fraction of water of each sample was achieved by evaporating water in a vacuum chamber or through blow-drying in air at approximately 40 °C. The compositions of the final samples are given in Figure 1. Samples of the third sequence were prepared identically with those of the second sequence, with the sole difference being the substitution of lysozyme with myoglobin.

It is important to note that the weight fractions of water we give in this article are added water to the purchased disaccharides and proteins. For all samples, except those containing trehalose, these water fractions are the true water fractions since both sucrose and the proteins were dry. However, this is not the case for trehalose since it was purchased in its dihydrated form, i.e., it contained 36 g of water per 378 g of dihydrated trehalose, giving 9.5 wt % water, which was not taken into account in the given weight fractions of water.

### DSC Measurements

DSC measurements were performed to monitor the crystallization of water, the glass transition of the protein–sugar–water mixture, and the denaturation temperature of the protein. The measurements were performed on a Q2000 differential scanning calorimeter (TA Instruments), and the samples were placed in a hermetic aluminum pan. The samples were cooled at a rate of 30 °C/min from 25 to −130 °C or −150 °C and then heated at a rate of 10 °C/min to 98 °C. Lastly, they were cooled back to 25 °C at the same cooling rate. *T*_g_ was determined from the inflection point of the glass transition and *T*_den_ from the minimum of the endothermic denaturation dip.

## Results

**Figure 2:**
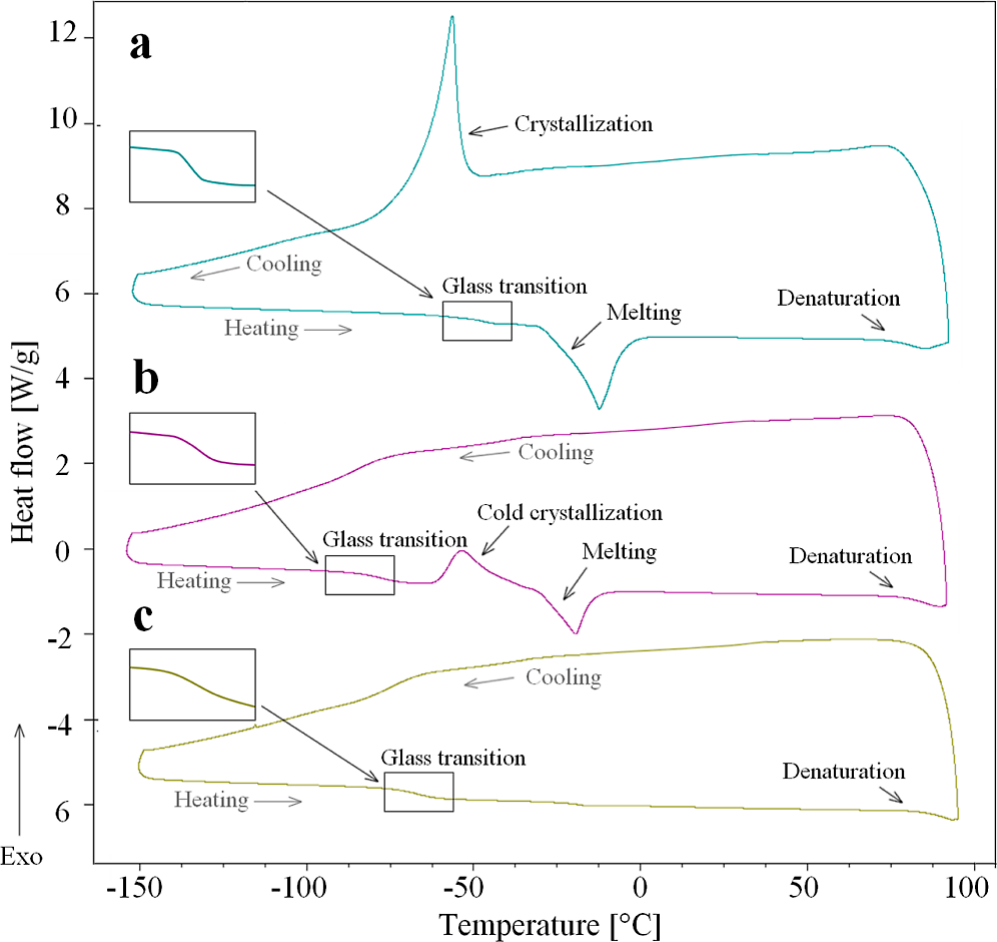
Three characteristic DSC curves showing the heat flow as a function of temperature, where (a) crystallization occurs during cooling, (b) crystallization occurs during heating (cold crystallization), and (c) no crystallization occurs. The concentrations for (a) are 45.7 wt % sucrose, 15.2 wt % lysozyme, and 39.1 wt % water; for (b) are 52.5 wt % sucrose, 17.5 wt % lysozyme, and 30.0 wt % water; and for (c) are 55.5 wt % sucrose, 18.5 wt % lysozyme, and 26.0 wt % water. For (a,b), melting is seen as an endothermic dip. The inset shows magnified images of the glass transition step. The protein denaturation is recognized as an endothermic dip at higher temperatures. The cooling and heating rates were 30 °C/min and 10 °C/min, respectively. (a) Displaced 6 W/g and (c) displaced −5 W/g.

The results from the DSC measurements can be divided into three different categories depending on the crystallization behavior. Figure 2a shows the typical behavior of a DSC cooling and heating cycle for samples with relatively high water concentrations (typically above 30–40 wt % water), where crystallization of water occurs during cooling (category *a*) and is recognized as a dramatic exothermic peak. At intermediate water contents (approximately 0–5 wt % lower than the threshold to obtain crystallization during cooling), no crystallization occurs during cooling (category *b*). However, cold crystallization during heating is present and is shown as an exothermic peak in Figure 2b. Finally, at the lowest water contents, crystallization occurs during neither heating nor cooling (category *c*), as shown in Figure 2c. The glass transition and protein denaturation are present during the heating cycle in all three cases, where the former is recognized as a small step at lower temperatures (see the inset) and the latter as an endothermic dip at higher temperatures. In addition, the melting is characterized as an endothermic dip during the heating cycle and is present for categories *a* and *b*. For category *c*, no melting can be detected as a consequence of no crystallization. These differences in the crystallization behavior cause large differences in how the investigated systems behave at low temperatures, as evident in the section “Effect on glass transition temperature” where we discuss how *T*_g_ depends on the water content. More experimental results are presented in Figures S1 and S2 of the Supporting Information for some of the other investigated samples. Furthermore, in Figures S3 and S4, we show how different characteristics about *T*_g_ and the ice melting *T*_m_ are obtained, and in Tables S1–S7, we present the so-obtained values of the analysis together with values of *T*_den_.

### Synergistic Effects of Trehalose and Sucrose

**Figure 3:**
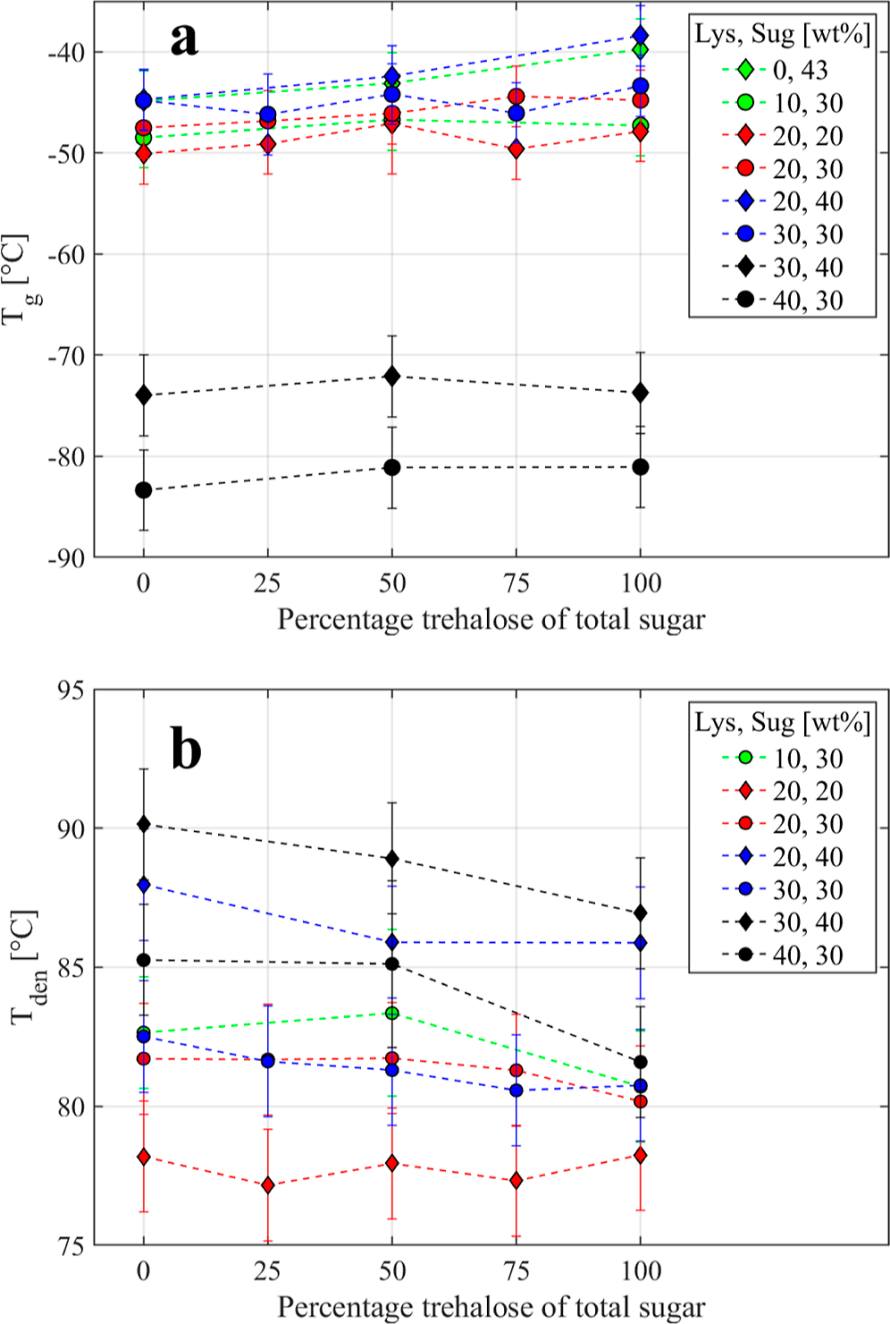
(a) *T*_g_ and (b) *T*_den_ as a function of the sucrose-to-trehalose weight ratio. The legend displays the variation in wt % of lysozyme and the total amount of sugar in the samples.

Trehalose and sucrose are notable for their stabilizing effects on proteins, which are critical in maintaining protein structure and functionality under stress conditions. The *T*_g_, which reflects the thermal stability of the protein–sugar–water matrix, is influenced by these sugars, potentially enhancing the overall stability of the protein. In addition, the presence of these sugars can significantly raise the denaturation temperature of a protein, which is another crucial measure of its thermal stability. The *T*_g_ and *T*_den_ as a function of the sucrose-to-trehalose weight ratio can be seen in Figure 3a,b, respectively. Both *T*_g_ and *T*_den_ change linearly (within the experimental errors) when one of the disaccharides is partially replaced by the other. Thus, samples with a mixture of trehalose and sucrose behave as a linear combination of the two corresponding samples with a single disaccharide, which implies that no synergistic effects occur when the two disaccharides are mixed. Figure 3a also reveals that in samples undergoing crystallization (the cluster of data points in the temperature range of −50 to −40 °C), the *T*_g_ is considerably higher than that for the fully amorphous samples (the data points below −70 °C). The reason for this is that when the water content is sufficiently high for initiating ice formation, the concentration of amorphous water becomes lower than that at the water concentration just below the concentration where ice starts to form. Thus, at a water concentration below this “ice nucleation point”, the sample is in a metastable state with more amorphous water than above the “ice nucleation point”, where the sample is a freeze-concentrated solution with additional ice. Since *T*_g_ decreases with an increasing amount of amorphous water, *T*_g_ becomes considerably lower just below the “ice nucleation point” than just above it. Figure 3a also shows that samples with a higher trehalose content exhibit a slightly higher *T*_g_. This is clearly seen for the partially crystalline samples and would also be evident for the fully amorphous samples if we had taken the water from the dihydrated trehalose into account. Thus, the trehalose-containing samples exhibit a slightly higher *T*_g_ than the corresponding sucrose-containing samples. For the *T*_den_ (see Figure 3b), the trend is opposite and *T*_den_ typically decreases slightly with increasing trehalose concentration.

### Maximum Water Content before Crystallization

**Figure 4:**
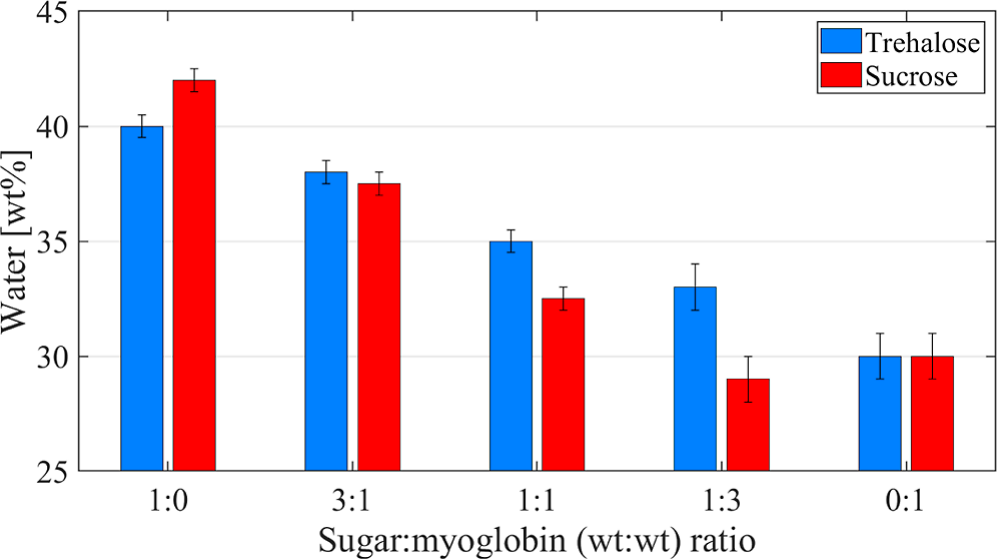
Maximal water content before crystallization occurs during cooling for different weight ratios of sugar-to-protein. The blue and red bars represent the trehalose- and sucrose-containing systems, respectively. The results presented for the trehalose-containing systems were obtained from ref (26).

The maximum water content of a sample before crystallization occurs is an effective measure of its hydration water. The tendency for crystallization is higher in bulk-like water, while water molecules interacting with the protein and/or sugar molecules remain in an amorphous state below the *T*_g_. Assuming both protein and sugar remain fully hydrated when the sugar-to-protein ratio varies, the maximum content of amorphous water in the system should be a weighted average of the binary systems. This assumption is confirmed by the data presented in Figure 4 for the trehalose-containing systems, suggesting that basically no trehalose molecules replace water molecules at the protein surface in the case of myoglobin. If trehalose molecules were to replace water molecules at the protein surface, then the trend would not be linear. This finding was obtained already in ref (26) and thereafter almost fully confirmed by neutron diffraction and structural modeling, where only a few trehalose molecules interact directly with the protein surface.^39^ Thus, the results for the trehalose-containing samples are in perfect agreement with the preferential hydration model.^31^ However, as shown in Figure 4, the same trend is not observed for the sucrose-containing systems, as the maximum amount of amorphous water is considerably less for intermediate protein–sugar compositions, showing that the protein and sucrose molecules are not able to maintain their full hydration as in the binary systems. Instead, the results imply that a significant amount of sucrose replaces water at the protein surface, thereby lowering the total hydration of both protein and the sucrose molecules. However, the replacement of water at the protein surface is not substantial enough to agree with the water replacement model.^37^ Rather, the results should be considered consistent with a substantially less pronounced preferential hydration model, in qualitative agreement with neutron diffraction and structural modeling data presented in ref (28).

### Effect on Glass Transition Temperature

**Figure 5:**
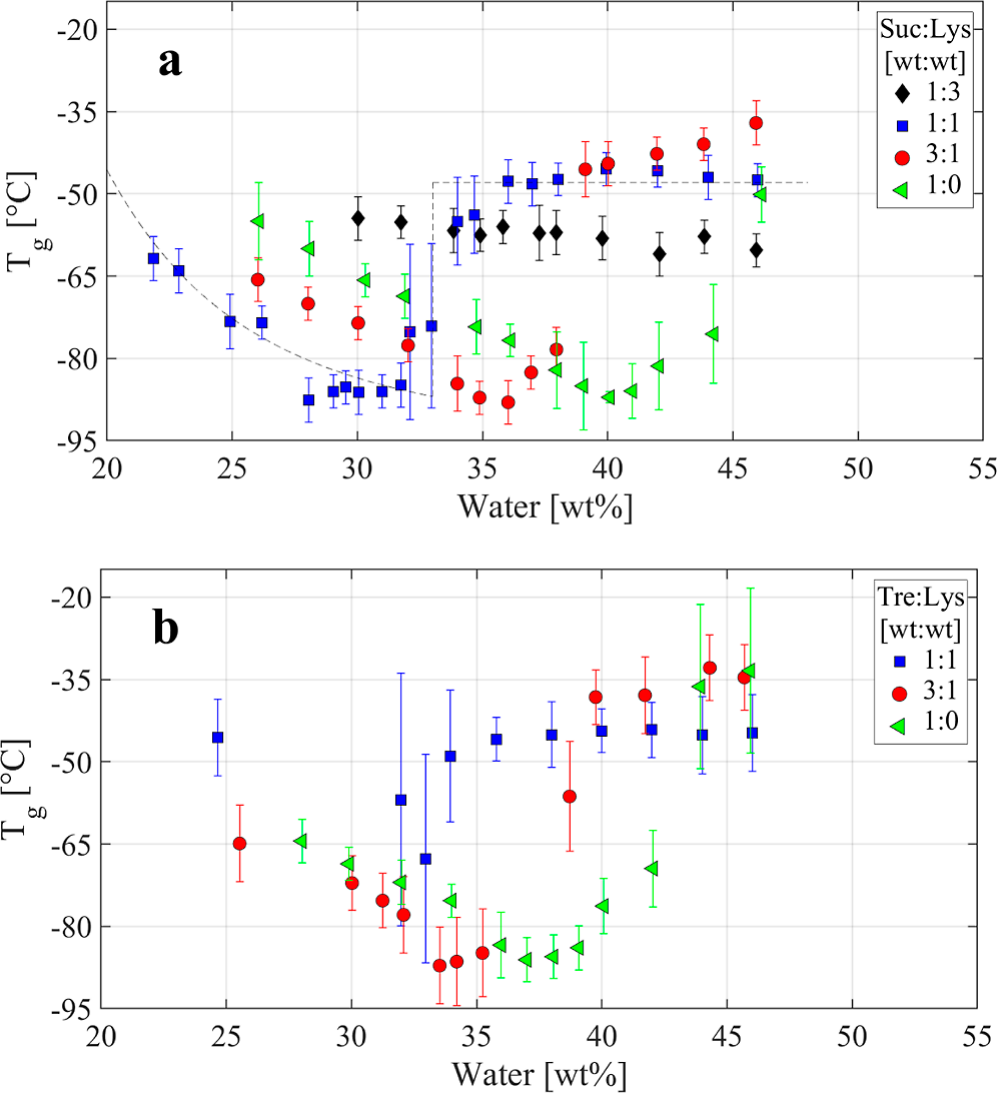
*T*_g_ as a function of the wt % water in the samples with (a) sucrose and (b) trehalose. The symbols for the different sugar to protein ratios are given in the figure legends. The dashed line in (a) illustrates the typical behavior for aqueous solutions, where *T*_g_ first decreases with increasing water content due to a plasticizing effect of water and thereafter increases abruptly to a stable value when crystallization occurs and a freeze-concentrated solution is obtained.

As previously mentioned, the water in the studied systems can be categorized as hydration water, which remains amorphous at all temperatures, or bulk-like water, which crystallizes at low temperatures. The former interacts sufficiently strongly with the protein or sugar to perturb its structure enough to avoid crystallization. It is only the amount of this amorphous water that strongly influences the *T*_g_ of the system. Figure 5a,b demonstrates how the *T*_g_ rapidly decreases with an increasing water content at low water concentrations (in Figures S5–S8 of the Supporting Information, we show the same data but plotted as a function of sugar or protein concentration). This indicates that a greater amount of hydration water accelerates the dynamics of the protein and sugar molecules. However, this trend changes abruptly at higher water contents, where partial crystallization occurs during cooling (see the dashed line in Figure 5a). In these cases, a freeze-concentrated solution forms alongside ice. These solutions have less amorphous (hydration) water compared to the samples with intermediate water contents, such as those displayed in Figure 2b. Consequently, their *T*_g_ is higher and becomes relatively unaffected by further variations in the water content. The reason is that only the amount of ice changes with the water concentration in this high water content regime.

In the case of the pure sugar solutions without lysozyme, the *T*_g_ values presented in Figures 3a and 5 can be compared with literature values presented in ref (40) for sucrose solutions and ref (41) for trehalose solutions. In the case of sucrose, the agreement is very good, except at water concentrations close to the “ice nucleation point”. It is clear that we have managed to reach higher concentrations of water before crystallization occurs, compared to the data presented in ref (40). The reason for this is most likely that we have used a faster cooling rate (30 °C/min down to −130 °C) than in previous studies and thereby been able to avoid crystallization of water for concentrations up to 40 wt %. A previous study^42^ of sucrose–water solutions has shown that both amount of amorphous water and the melting temperature of formed ice depend on the cooling and heating rates. Since we have reached higher concentrations of amorphous water with our fast cooling, we also reached lower *T*_g_ values (down to −88 °C). The results for trehalose become similar to ref (41) if we count the water in the dihydrated trehalose and only compare the data up to water concentrations of about 35 wt %. At the highest water concentrations, we obtain a substantially lower *T*_g_ and as for sucrose, we are able to reach a higher water concentration before crystallization occurs [the lowest *T*_g_ (−86 °C) is obtained at 37 wt % water without the dihydrate water counted, which implies 43 wt % water with that water included].

In the high water concentration region where the solutions become freeze concentrated, we obtain a *T*_g_ value (denoted as *T*_g_′ in ref (40)) of about −49 °C for the freeze-concentrated sucrose solutions containing 46 and 57 wt % water (see Figures 3a and 5a), which can be compared to the value −46 °C given in ref (40). Also, for the freeze-concentrated sucrose solutions containing lysozyme, *T*_g_ values of −45 to −50 °C are obtained for moderate protein concentrations (see Figure 3a). For the freeze-concentrated trehalose solutions, we obtain *T*_g_ values in the range of −33 to −40 °C (see Figures 3a and 5b). In this case, literature values in the range of −22 to −40 °C have been reported,^41^ with values around −30 °C as the most common ones. For the freeze-concentrated samples containing both trehalose and lysozyme, *T*_g_ values in the range of −48 to −32 °C are obtained, as shown in Figures 3a and 5b.

In Tables S1–S7 of the Supporting Information, we present the characteristics of *T*_g_, *T*_den_, and *T*_m_ for all of the sample compositions (except those containing both sucrose and trehalose) we have measured. The tables show that the step in heat capacity (Δ*C*_p_) depends on the composition and decreases slightly with both increasing protein concentration and decreasing concentration of amorphous water. The same decreasing trend with increasing protein concentration was also observed in ref (43). However, in ref (43), the lysozyme–sucrose samples were almost completely dry and therefore the protein did not contribute to the Δ*C*_p_ since proteins need water (or at least a solvent) to exhibit glass transition-related dynamics.^33,34^ Thus, the quantitative differences between our findings and the results obtained in ref (43) are expected.

Another interesting observation in Tables S1–S7 of the Supporting Information is that the onset temperature of ice melting *T*_m_ (denoted *T*_m_′ in ref (40)) is located at a considerably lower temperature (typically around 30 °C lower) than the endothermic main dip due to ice melting. The reason that some of the ice begins to melt at such low temperatures is that this ice is located in small nanometer-sized ice clusters, which exhibit a substantially depressed melting temperature. In fact, the ice melting may begin at such a low temperature that it overlaps with *T*_g_ and therefore makes the calculated Δ*C*_p_ of *T*_g_ larger than it should be, as indicated for some of the samples in Tables S1–S7.

### Effect on Denaturation Temperature

**Figure 6:**
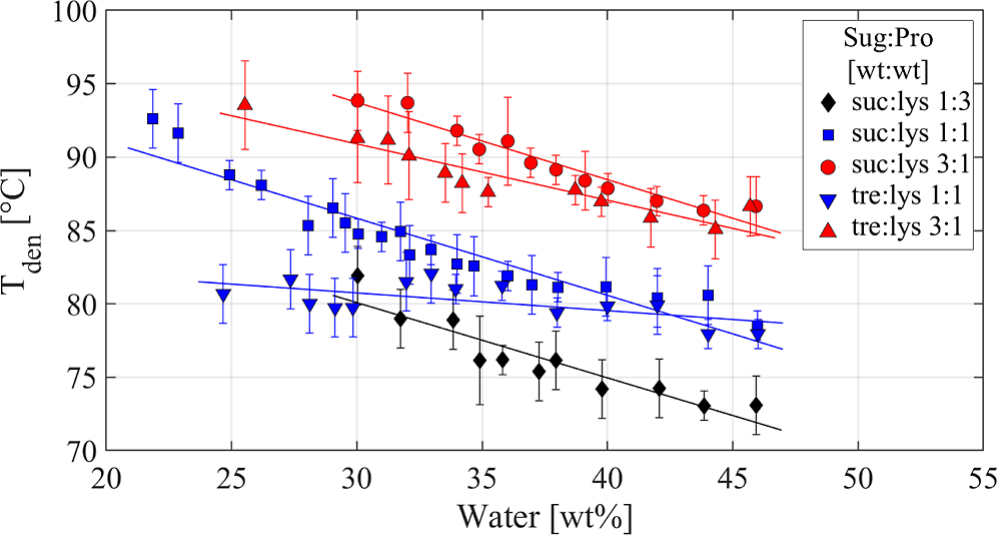
*T*_den_ as a function of water content (wt %). The black diamonds, blue squares, and red circles represent sucrose-containing systems with weight ratios of 1:3, 1:1, and 3:1 between sucrose and lysozyme. The inverted blue triangles and red triangles denote trehalose-containing systems with weight ratios of 1:1 and 3:1 between trehalose and lysozyme.

Figure 6 illustrates that *T*_den_ decreases with increasing water content. Unlike the case with *T*_g_, high temperatures leave the samples unaffected by crystallization. This suggests that the *T*_den_ consistently decreases across the entire concentration range as the water content increases. However, by comparing the behavior of the sucrose- and trehalose-containing samples, it is evident that there is a stronger dependence for the samples with sucrose, particularly at low sugar-to-protein ratios (see also Figures S9–S12 of the Supporting Information). The most striking finding for all samples is, however, that *T*_den_ increases dramatically with increasing amounts of sugar relative to the amount of the protein, suggesting that this ratio is crucial for the thermal stability of the protein close to its denaturation temperature.

## Discussion

The present results have shown that there is a clear difference in how trehalose and sucrose interact with the proteins and that these differences may have implications for *T*_g_ and *T*_den_. The data in Figure 4 demonstrate that, below the *T*_g_, both myoglobin and trehalose molecules are equally hydrated in the sense that they are surrounded by a similar amount of amorphous water, as observed in their respective two-component systems. Thus, there is no evidence to suggest that trehalose molecules displace water molecules at the protein surface, thereby reducing the total amount of hydration water or the confined water that does not crystallize at any temperature. However, the same cannot be seen when sucrose is added to an aqueous solution of myoglobin. As indicated by the data in Figure 4, the presence of sucrose leads to a reduction in the amount of amorphous water below the *T*_g_. This decrease could be due to either clustering of sucrose and myoglobin or, more likely, a direct interaction between the two. Thus, an estimation of the amount of amorphous water provides important structural information and shows that the trehalose- and sucrose-containing systems must have clear structural differences in the solvent surrounding the protein. However, the question is how these structural differences influence the stability of the protein. In the case of *T*_g_, the data presented in Figures 5 and S5–S8 in the Supporting Information show that *T*_g_ is higher for the trehalose-containing samples compared to the corresponding sucrose-containing samples (particularly if we count the water in the dihydrated trehalose). This finding is consistent with results from both quasielastic neutron scattering^28^ and dielectric spectroscopy,^44^ showing that the protein and solvent dynamics are slower for the trehalose-containing samples compared to the corresponding sucrose-containing samples. The reason for this is most likely that the *T*_g_ values of pure trehalose and highly concentrated trehalose solutions are higher than those for the corresponding sucrose samples,^45^ but the finding that trehalose is more excluded from the protein surface may also contribute to a higher protein stability at temperatures around *T*_g_.

In the case of *T*_den_, the values are identical (within the experimental errors) for samples with sucrose and trehalose at high water concentrations around 45 wt %, as seen in Figure 6. However, in the case of sucrose, the *T*_den_ increases more rapidly, compared to the case of trehalose, with decreasing water content, leading to a significantly higher *T*_den_ at low water contents, as illustrated in Figure 6. Also, in this case, a plausible explanation is the displacement of water molecules at the protein surface by sucrose molecules (although this led to a possible decrease of *T*_g_ in contrast to the observed increase of *T*_den_), an effect that possibly becomes more pronounced at relatively low water concentrations. In favor of this hypothesis is the finding by neutron diffraction and structural modeling that the preferential hydration effect is only slightly weaker for sucrose compared to trehalose for samples of 50 wt % water.^28^ Thus, the structural differences between the sucrose- and trehalose-containing systems seem to be small at high water concentrations, but the difference increases with decreasing water content. However, it should be noted that in the case of the *T*_g_ for the freeze-concentrated solutions, the direct binding of sucrose to the protein might be substantial even at high water concentrations, since a large fraction of this water is then located in bulk-like ice domains.

For the trehalose-containing samples, the *T*_den_ is only weakly dependent on the water concentration (at least at a sugar-to-protein ratio of 1:1), as shown in Figure 6, despite the fact that the macroscopic viscosity should decrease with increasing water content due to the common plasticizing effect water has on disaccharides and other solutes.^46^ The reason is most likely that the local environment around each protein molecule remains almost unchanged (for the water concentrations used in this study) due to preferential hydration, which maintains the water hydration layer and thereby also the local microscopic viscosity around each protein molecule. However, it should be noted that *T*_den_ increases substantially with increasing trehalose-to-protein ratios. This occurs despite the maintained water-hydration layer and the potential decrease in macroscopic viscosity. The reason for this is that with more trehalose molecules per protein molecule, the protein hydration layer becomes more fully surrounded by trehalose molecules. Thus, although the trehalose molecules are generally not binding to the protein surface, they are still stabilizing the protein via the hydration layer, e.g., by slowing down the dynamics of this protein hydration layer.^39^

In the case of samples containing sucrose, the situation appears different; it seems that an increasing number of sucrose molecules directly bind to the protein as the water content decreases. In this way, the local microscopic viscosity around each protein molecule follows more closely the change in the macroscopic viscosity with the water concentration. Although, in this case, the protein is stabilized considerably more efficiently by an increase in the sucrose-to-protein ratio, even if it does not increase the macroscopic viscosity. It is also possible that the direct binding of sucrose to the protein surface leads to steric stabilization of the protein. Such an effect should also increase with an increasing sucrose-to-protein ratio. However, it should here be noted that our finding that *T*_den_ is higher for the sucrose-containing samples than for the corresponding trehalose-containing samples, at least at lower water concentrations, has not been observed in all previous studies.^30,47^

For instance, Hédoux et al.^30^ found that at a very high water concentration and a sugar-to-lysozyme ratio of 2:3, the *T*_den_ was higher for the sample with trehalose compared to the corresponding sample with sucrose. This finding is consistent with our data, where an extrapolation to very high water concentrations also gives a higher *T*_den_ value for trehalose. On the other hand, at completely dry conditions when lysozyme unavoidably must interact directly with the sugar, trehalose again gave a higher *T*_den_ than sucrose.^47^ These previous findings suggest that trehalose is a more efficient stabilizer of the native protein structure than sucrose, both at very high water concentrations when it is likely that the protein is fully hydrated by water irrespective of the sugar and at completely dry conditions when hydration water is lacking for both sugars. Thus, it seems that when the structures around the protein molecules are similar for the two disaccharides, trehalose is a more efficient protein stabilizer. However, at the intermediate water concentrations used in this study, there are structural differences between the trehalose- and sucrose-containing systems (i.e., a less pronounced preferential hydration effect for sucrose), and these differences seem to be in favor of sucrose in the case of stabilizing the native protein structure. It is not clear why preferential hydration seems to be detrimental for protein stability at high temperatures around the *T*_den_, since the thermodynamical implication of the excluded volume effect is that preferential hydration stabilizes the native state of the protein.^6,35,36^ Perhaps the reason for the deviation from the thermodynamical prediction is that the hydrogen bonding to the protein surface plays a weaker role at such high temperatures and the protein is instead more sterically stabilized by the sugar. This further implies that sucrose cannot bind directly to the protein in a similar way as established denaturants, such as urea and guanidinium chloride, do, where they replace all water molecules, even interior water molecules which stabilize the native state of the protein by hydrogen bonding between different amino acids in the protein backbone.^48,49^ This is obviously not the case for sucrose, which likely interacts only weakly with the protein (at least at temperatures close to *T*_den_) without displacing interior water molecules. However, even if sucrose binding to the protein surface is not causing the same detrimental effects as well-known denaturants, such binding should still reduce the excluded volume effect^6,35,36^ and thereby potentially destabilize the native state of the protein. It is therefore not obvious that the somewhat more pronounced binding of sucrose to the protein surface, compared to trehalose, should be beneficial for the protein stability, but at high temperatures close to *T*_den_, other effects, such as steric stabilization, seem to be more important. This also implies that there is no general relation between *T*_g_ and *T*_den_ as previously suggested.^24,25^ This is an important finding since it indicates that the exact stabilization mechanism can be complex and can vary with both temperature and the water concentration. We propose that the protein stability at low temperatures around *T*_g_ is mainly governed by the protein dynamics (which is caused by the solvent dynamics^33,34^), whereas *T*_den_ is mainly determined by thermodynamics and steric stabilization.

Due to the slightly different effects of sucrose and trehalose, it is possible that the two disaccharides enhance the stability of proteins in slightly different ways and that they therefore complement each other and cause a positive synergistic effect when they are mixed. This was investigated by partly replacing one of the disaccharides with the same amount of the other. However, the results presented in Figure 3 do not indicate that there is any such synergistic effect, since both *T*_g_ and *T*_den_ vary with the ratio between trehalose and sucrose as a linear combination of the two corresponding samples containing only one of the disaccharides. This finding further suggests that the two disaccharides behave (i.e., interacting with protein, water, and other sugar molecules) exactly the same when they are mixed, as they do in the corresponding systems with a single disaccharide. It also indicates that the stabilization mechanism is similar for the two disaccharides, although not necessarily identical.

## Conclusions

In this study, we have compared the protein-stabilizing properties of the two disaccharides, trehalose and sucrose. From the results, it is clear that both disaccharides exhibit strong stabilizing effects. However, it is unclear which is the most efficient stabilizer. The glass transition temperature, *T*_g_, of trehalose and its concentrated aqueous solutions is higher than that of sucrose, which seems to be advantageous for protein stability, at least at lower temperatures around the *T*_g_. On the other hand, the results show that sucrose to a greater extent binds directly to the protein surface, which appears to be beneficial for maintaining the protein in its native state. This suggests that trehalose’s more pronounced preferential hydration effect could be advantageous at lower temperatures, where the hydrogen bonds are stronger in relation to the thermal energy. However, at higher temperatures, sucrose’s less pronounced preferential hydration effect might be more advantageous, possibly due to a steric stabilization of the native protein structure. This further implies that the mechanism for protein stabilization might be somewhat different at low and high temperatures (and at different concentrations of the three components: protein, sugar, and water) and that it also may vary between different proteins depending on their surface properties, such as hydrophilicity and hydrophobicity. Generally, it seems likely that the thermodynamics is more important for the stability of the native state of the protein, whereas the stability in the glassy state is governed by the internal relaxation dynamics. Finally, despite the fact that the two disaccharides interact slightly differently with the protein, we could not detect any positive synergistic effect by mixing trehalose and sucrose. Instead, both *T*_g_ and *T*_den_ vary with the ratio between trehalose and sucrose as a linear combination of the two corresponding samples containing only one of the disaccharides.

## Supporting Information Available

Additional DSC data, characteristics of the glass transition, denaturation, and melting processes; and variation of glass transition temperature and denaturation temperature with the sugar and protein concentrations (PDF)
