# Supplementary material for: Comparison of Sucrose and Trehalose for Protein Stabilization Using Differential Scanning Calorimetry
Source: J Phys Chem B. 2024 May 11;128(20):4922–30. doi: 10.1021/acs.jpcb.4c00022 (PMC11129304; doi:10.1021/acs.jpcb.4c00022)
Supplement: Supplementary file 1 — jp4c00022_si_001.pdf [file jp4c00022_si_001.pdf]

A Comparison of Sucrose and Trehalose for Protein Stabilization using Differential Scanning Calorimetry -Supplementary Information

Olivia Jonsson, Agnes Lundell, John Rosell, Sophie You, Kajsa Ahlgren, and Jan Swenson*

Department of Physics, Chalmers University of Technology, Gothenburg SE-412 96, Sweden.

E-mail: jan.swenson@chalmers.se

Contents

1 Differential Scanning Calorimetry cycles **S2**

2 Tables of experimentally obtained data **S6**

3 Additional plots glass transition temperature **S14**

4 Additional plots denaturation temperature **S17**

1 Differential Scanning Calorimetry cycles

Figures S1 and S2 present representative DSC curves for the three distinct categories. The left panel illustrates the cycles of systems that experience crystallization upon cooling, attributed to the high water content. The central panel depicts the cycles of systems that do not crystallize during cooling but undergo cold crystallization during the heating cycle. Finally, the right panel displays the systems that exhibit no crystallization events.

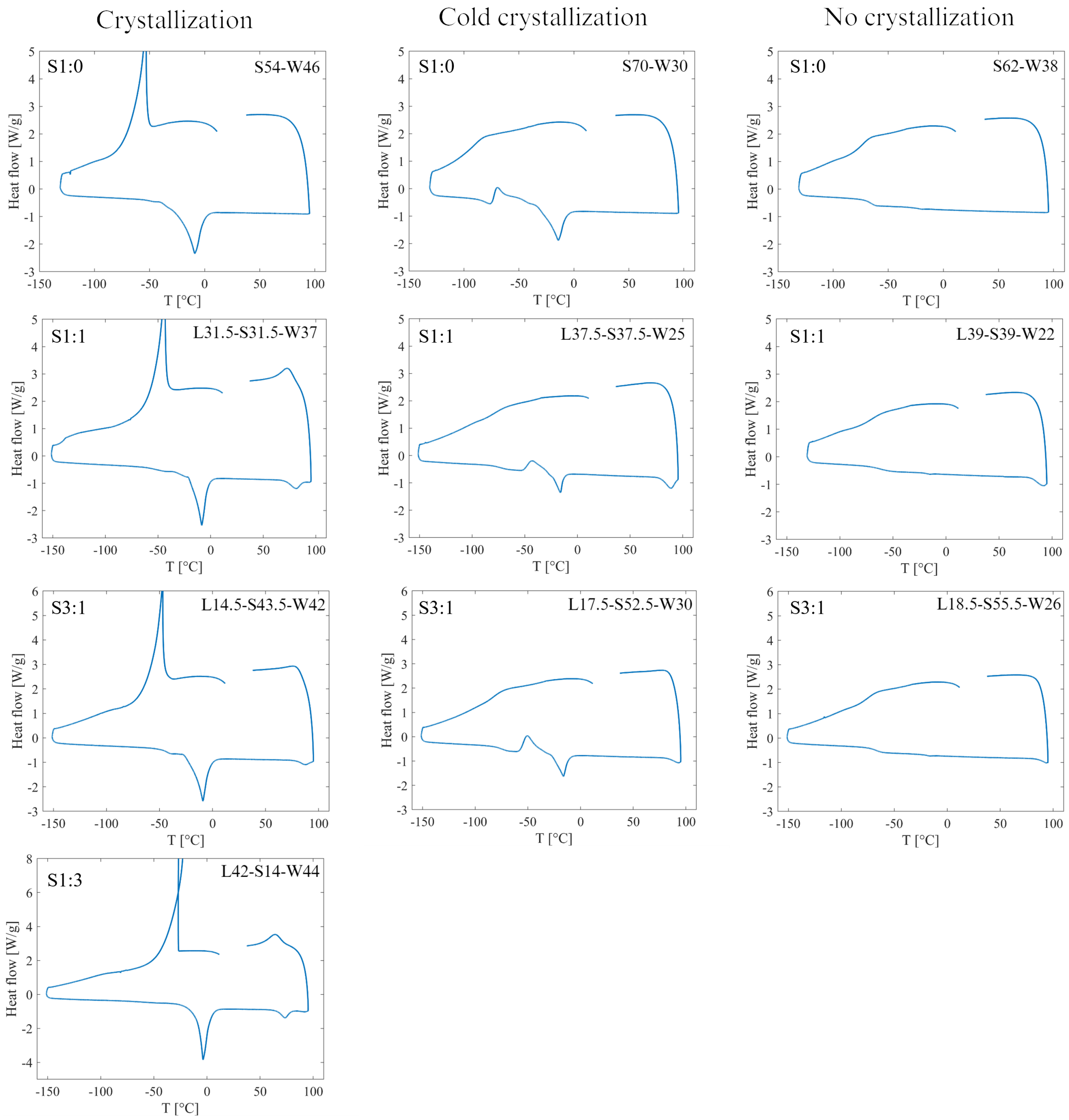

Figure S1: Representative DSC cycles for sucrose containing systems at different sucrose:lysozyme ratios as well as different levels of hydration.

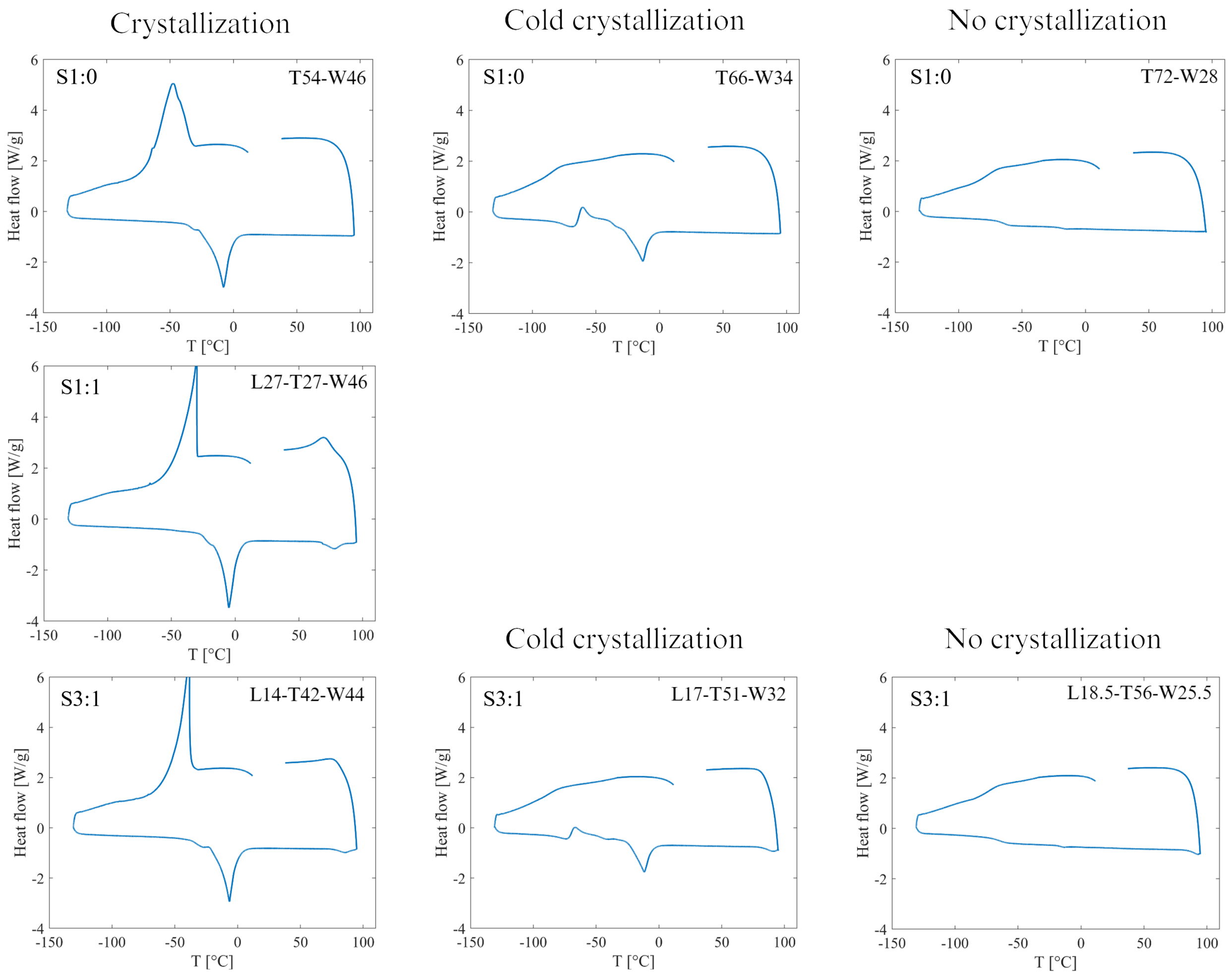

Figure S2: Representative DSC cycles for trehalose containing systems at different trehalose:lysozyme ratios as well as different levels of hydration.

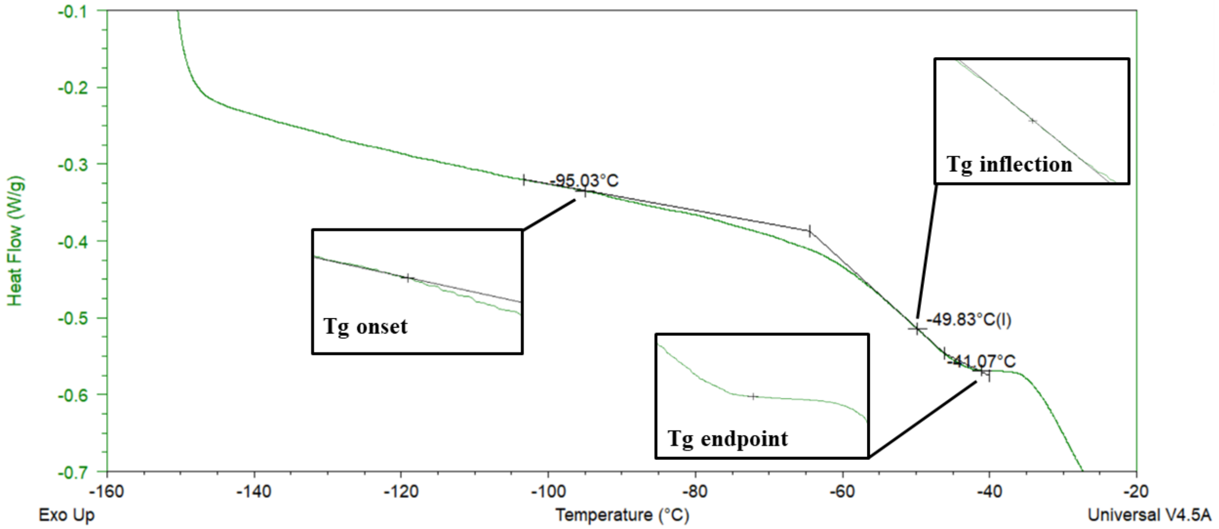

Figure S3: Demonstration of how a typical glass transition was analysed. The insets show the glass transition onset, inflection, and endpoint.

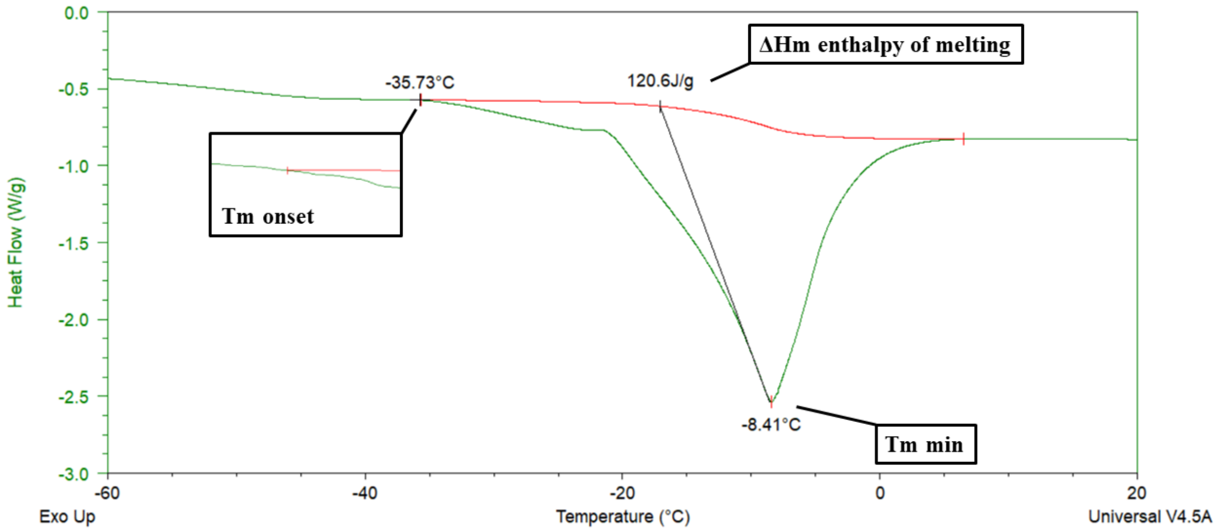

Figure S4: Demonstration of how a typical melting process was analysed. The insets point out the onset and melting temperatures, as well as the enthalpy of the melting process.

2 Tables of experimentally obtained data

Tables S1-S7 present a summary of all experimentally obtained data including the wt% of each component in the samples. The change in heat capacity (ΔCp) during the glass transition as well as the onset (T_g^{onset}), inflection (T_g^{infl}) and end (T_g^{end}) points of the glass transition are presented. In addition, the denaturation temperatures (T_{den}) are listed for the samples that contain protein. Whether the sample undergo crystallization, cold crystallization, or both is also stated. For these samples the onset (T_m^{on}) and melting temperature (T_m) (i.e. the position of the endothermic dip) of the melting process, as well as the melting enthalpy (ΔH_m) were recorded, from which the wt% amorphous water in the system (Am. H_2O) was calculated.

Table S1: Trehalose:Lysozyme 3:1

Tre [wt%]	Lys [wt%]	H_2O [wt%]	ΔC_p [J/g/K]	T_g^{onset} [°C]	T_g^{infl} [°C]	T_g^{end} [°C]	T_{den} [°C]	T_m^{on} [°C]	T_m [°C]	ΔH_m [J/g]	Am. H_2O [wt%]	Cryst.	Cold cryst.
56.07	18.41	25.53	1.9±0.3	-97.8±17	-64.9±7	-54.8±7	-93.5±3	-	-	-	25.5	No	No
52.68	17.30	30.02	1.7±0.3	-91.4±10	-72.1±5	-61.4±6	91.3±3	-	-14.3±3	-	30.0	No	Yes
51.76	16.99	31.24	1.5±0.3	-102.1±10	-75.3±5	-69.7±7	91.2±3	-35.9±5	-12.3±1	42.4±10	21.2	No	Yes
51.13	16.79	32.08	1.2±0.5	-110.4±7	-77.9±7	-74.2±5	90.1±3	-36.2±4	-11.6±2	47.3±7	20.9	No	Yes
50.05	16.43	33.52	0.8±0.4	-110.8±6	-87.2±7	-81.9±7	88.9±2	-36.3±4	-11.4±1	57.7±5	19.6	Yes	Yes
49.54	16.26	34.20	0.9±0.4	-112.9±6	-86.4±8	-81.6±8	88.2±2	-35.9±4	-10.7±2	62.0±6	19.2	Yes	Yes
48.76	16.01	35.23	0.6±0.4	-98.6±7	-84.9±8	-78.3±10	88.7±1	-35.9±4	-10.4±1	62.0±5	20.5	Yes	Yes
45.99	15.3	38.71	1.1±0.8	-91.5±20	-56.3±10	-49.6±17	87.8±1	-33.9±5	-8.7±1	72.3±11	21.8	Yes	Yes
45.21	15.04	39.75	1.6±0.7	-80.4±15	-38.2±5	-35.5±10	87.0±1	-31.5±5	-7.5±2	74.0±5	22.6	Yes	No
43.58	14.7	41.72	1.9±0.8	-58.3±12	-37.9±7	-34.4±10	85.9±2	-31.1±5	-7.3±1	83.8±10	22.2	Yes	No
41.64	14.04	44.31	1±0.8	-66.3±15	-32.9±6	-41.4±13	85.1±2	-28.5±8	-6.5±1	80.2±5	26.7	Yes	No
40.62	13.7	45.69	-	-75.1±20	-34.7±6	-40.9±13	86.6±2	-23.2±	-6.1±2	82.2±8	28.0	Yes	No

Table S2: Trehalose:Lysozyme 1:1

Tre [wt%]	Lys [wt%]	H_2O [wt%]	ΔC_p [J/g/K]	T_g^{onset} [°C]	T_g^{infl} [°C]	T_g^{end} [°C]	T_{den} [°C]	T_m^{on} [°C]	T_m [°C]	ΔH_m [J/g]	Am. H_2O [wt%]	Cryst.	Cold cryst.
37.61	37.72	24.67	1.3±0.4	-59.9±15	-45.6±7	-41.5±7	-80.7±2	-	-	-	-	-	-
34.03	34.01	31.97	0.8±0.5	-92.8±11	-56.9±23	-50.8±8	81.5±2	-38.8±6	-8±2	63.0±5	16.1	Yes	No
33.27	33.27	32.97	0.6±0.5	-95.7±8	-67.7±19	-55.3±17	82.1±2	-36.7±2	-7.6±5	64.4±4	17.1	Yes	No
33.01	33.05	33.94	1.5±0.6	-77.5±20	-49.0±12	-41.4±4	81.0±1	-35.4±4	-7.0±1	71.2±7	16.0	Yes	No
32.08	32.12	35.80	1.4±0.6	-72.2±9	-45.9±4	-41.6±4	81.2±1	-33.0±6	-5.9±2	73.3±11	17.8	Yes	No
30.96	31.05	37.98	1.5±0.6	-68.2±10	-45.1±6	-41.4±4	79.4±1	-32.4±5	-6.5±2	88.7±5	15.6	Yes	No
29.98	30.02	40.00	0.8±0.4	-66.1±15	-44.4±4	-41.7±3	79.8±1	-32.1±4	-5.7±4	95.5±2	16.0	Yes	No
28.96	29.04	41.99	0.5±0.5	-58.5±7	-44.2±5	-41.4±4	79.9±2	-34.5±6	-2.2±6	108.7±8	14.0	Yes	No
27.95	28.03	44.02	-	-57.6±7	-45.2±7	-41.7±7	78.0±1	-30.1±5	-4.1±2	116.9±8	13.9	Yes	No
26.96	27.04	46.00	0.7±0.5	-60.1±8	-44.8±7	-41.0±5	78.0±1	-31.2±4	-4.9±5	114.2±9	17.9	Yes	No

Table S3: Trehalose:Lysozyme 1:0

Tre [wt%]	Lys [wt%]	H_2O [wt%]	ΔC_p [J/g/K]	T_g^{onset} [°C]	T_g^{infl} [°C]	T_g^{end} [°C]	T_{den} [°C]	T_m^{on} [°C]	T_m [°C]	ΔH_m [J/g]	Am. H_2O [wt%]	Cryst.	Cold cryst.
71.98	0.0	28.02	1.7±0.3	-95.6±5	-64.5±4	-56.9±8	-	-	-	-	28.0	No	No
70.1	0.0	29.9	1.8±0.3	-91.6±3	-68.6±3	-60.6±4	-	-	-	-	28.0	No	No
68.01	0.0	31.99	1.7±0.2	-94.0±5	-72.0±4	-60.9±3	-	-	-14.0±1	-	32.0	No	Yes
66.00	0.0	34.0	1.6±0.2	-96.6±75	-75.3±3	-69.7±3	-	-	-13.2±1	-	34.0	No	Yes
64.02	0.0	35.98	0.9±0.4	-98.7±6	-83.4±6	-79.7±8	-	-34.4±5	-10.8±2	64.9±3	20.5	No	Yes
63.0	0.0	37.0	0.9±0.3	-110.7±41	-86.1±4	-82.1±6	-	-35.0±3	-11.1±1	74.1±8	19.0	No	Yes
61.94	0.0	38.06	0.8±0.2	-110.6±4	-85.5±4	-81.9±4	-	-35.5±4	-10.9±1	77.5±7	19.3	Yes	Yes
60.92	0.0	39.08	0.8±0.3	-110.9±10	-83.9±4	-80.1±6	-	-34.5±4	-10.6±1	79.7±6	20.0	Yes	Yes
59.93	0.0	40.07	0.7±0.2	-103.7±9	-76.3±5	-72.1±10	-	-33.1±3	-9.7±1	82.0±5	20.6	Yes	Yes
57.96	0.0	42.04	0.5±0.3	-93.5±7	-69.5±7	-63.3±5	-	-33.0±4	-8.8±1	86.8±3	21.7	Yes	Yes
56.07	0.0	43.93	0.4±0.3	-43.6±7	-43.6±15	-	-	-31.1±3	-8.6±1	92.7±3	22.4	Yes	No
54.08	0.0	45.92	-	-48.8±7	-48.8±15	-	-	-28.3±4	-7.8±1	92.3±10	25.3	Yes	No

Table S4: Sucrose:Lysozyme 1:0

Suc [wt%]	Lys [wt%]	H_2O [wt%]	ΔC_p [J/g/K]	T_g^{onset} [°C]	T_g^{infl} [°C]	T_g^{end} [°C]	T_{den} [°C]	T_m^{on} [°C]	T_m [°C]	ΔH_m [J/g]	Am. H_2O [wt%]	Cryst.	Cold cryst.
73.93	0.0	26.07	1.7±0.3	-88.1±7	-55.0±5	-47.6±3	-	-	-	-	26.07	No	No
71.91	0.0	28.09	1.9±0.3	-95.5±5	-60.0±4	-53.5±2	-	-	-	-	28.09	No	No
69.68	0.0	30.32	1.8±0.2	-95.0±3	-65.7±3	-58.8±2	-	-	-	-	30.32	No	No
68.11	0.0	31.89	1.8±0.2	-96.9±4	-68.7±4	-68.6±4	-	-	-	-	31.89	No	No
65.24	0.0	34.76	1.7±0.3	-95.6±5	-74.3±4	-67.3±3	-	-	-16.2±3	-	-	No	Yes
63.89	0.0	36.11	1.6±0.3	-98.8±3	-76.7±4	-70.3±2	-	-	-15.5±3	-	-	No	Yes
62.03	0.0	37.97	1.3±0.1	-103.7±7	-82.1±3	-77.3±2	-	-40.1±3	-14.1±3	-	-	No	Yes
60.98	0.0	39.02	1.0±0.4	-112.3±8	-85.0±5	-81.7±3	-	-39.4±3	-13.6±4	65.4±4	24.17	No	Yes
59.92	0.0	40.08	0.7±0.2	-109.1±1	-87.1±3	-84.0±2	-	-40.1±2	-11.4±2	69.6±3	24.3	No	Yes
59.02	0.0	40.98	0.6±0.3	-99.2±5	-86.0±4	-83.2±4	-	-38.2±3	-12.2±3	73.0±4	24.48	Yes	Yes
57.95	0.0	42.05	0.8±0.4	-106.8±8	-81.4±3	-77.7±2	-	-36.3±3	-11.6±2	76.8±3	24.76	Yes	Yes
55.77	0.0	44.23	0.4±0.3	-96.8±9	-75.6±8	-72.6±6	-	-37.2±4	-10.7±3	79.1±6	26.94	Yes	Yes
53.86	0.0	46.14	0.8±0.4	-78.4±5	-50.1±9	-45.4±6	-	-41.4±5	-9.0±3	92.3±5	25.57	Yes	No

Table S5: Sucrose:Lysozyme 3:1

Suc [wt%]	Lys [wt%]	H_2O [wt%]	ΔC_p [J/g/K]	T_g^{onset} [°C]	T_g^{infl} [°C]	T_g^{end} [°C]	T_{den} [°C]	T_m^{on} [°C]	T_m [°C]	ΔH_m [J/g]	Am. H_2O [wt%]	Cryst.	Cold cryst.
55.46	18.52	26.02	2.0±0.2	-100.4±4	-65.7±4	-52.4±7	-	-	-	-	26.0	No	No
53.95	18.02	28.03	1.7±0.1	-95.4±3	-70.0±3	-59.0±2	-	-	-	-	28.0	No	No
52.46	17.52	30.03	1.5±0.1	-94.6±2	-73.6±3	-64.3±4	93.8±2	-	-16.2±3	-	30.0	No	Yes
50.96	17.01	32.02	1.3±0.1	-97.5±3	-77.6±3	-69.9±3	93.7±2	-39.3±5	-14.8±2	35.8±3	23.9	No	Yes
49.46	16.55	33.99	0.7±0.2	-111.0±11	-84.6±5	-80.6±3	91.8±1	-38.1±4	-13.3±1	49.7±5	22.5	No	Yes
48.77	16.36	34.88	0.7±0.1	-114.4±5	-87.2±3	-83.0±3	90.5±1	-38.2±3	-12.6±2	54.9±6	22.1	No	Yes
47.99	15.99	36.02	0.6±0.2	-111.5±2	-88.0±4	-83.5±1	91.1±3	-38.2±4	-12.6±2	53.8±3	23.7	No	Yes
47.3	15.77	36.94	0.6±0.2	-103.8±8	-82.6±3	-77.3±4	89.6±1	-37.3±3	-11.4±2	62.6±3	22.4	Yes	Yes
47.04	15.76	37.95	0.6±0.3	-99.9±9	-78.4±4	-72.4±4	-89.1±1	-36.9±4	-10.7±2	64.1±4	23.0	Yes	No
45.67	15.23	39.1	1.2±0.2	-80.3±4	-45.6±5	-40.8±4	88.4±2	-36.8±5	-10.3±1	69.3±3	23.2	Yes	No
51.41	17.19	40.01	1.5±0.2	-95.1±5	-44.5±4	-40.0±3	87.9±1	-35.3±3	-9.6±2	65.1±3	23.0	Yes	No
43.52	14.54	41.96	1.7±0.1	-77.8±2	-42.7±3	-37.8±3	-87.0±1	-33.7±3	-9.0±1	73.7±3	25.5	Yes	No
42.11	14.06	43.83	1.9±0.4	-75.2±3	-41.0±3	-35.1±3	86.4±1	-32.4±3	-8.3±2	78.6±6	26.5	Yes	No
40.53	13.55	45.92	2.3±0.3	-76.1±4	-37.1±4	-30.2±3	86.7±2	-28.2±6	-7.2±2	83.3±3	27.9	Yes	No

Table S6: Sucrose:Lysozyme 1:1

Suc [wt%]	Lys [wt%]	H_2O [wt%]	ΔC_p [J/g/K]	T_g^{onset} [°C]	T_g^{infl} [°C]	T_g^{end} [°C]	T_{den} [°C]	T_m^{on} [°C]	T_m [°C]	ΔH_m [J/g]	Am. H_2O [wt%]	Cryst.	Cold cryst.
39.04	39.11	21.85	1.5±0.2	-90.4±4	-61.8±4	-42.5±3	92.61±2	-	-	-	21.9	No	No
38.54	38.6	22.86	1.4±0.2	-88.6±4	-64.0±4	-42.6±3	91.6±2	-	-	-	22.9	No	No
37.67	37.41	24.91	1.2±0.3	-95.8±5	-73.3±5	-53.1±3	88.8±1	-	-16.1±3	-	24.9	No	Yes
36.88	36.94	26.19	1.3±0.3	-95.9±3	-73.5±3	-61.2±4	88.1±1	-	-14.0±3	-	26.2	No	Yes
36.09	35.84	28.06	0.7±0.4	-97.8±3	-87.6±4	-82.5±4	85.3±2	-39.0±5	-12.6±1	40.1±5	18.2	No	Yes
35.53	35.42	29.05	0.7±0.1	-113.5±7	-86.0±3	-79.9±3	86.5±2	-38.6±5	-11.6±1	38.5±5	19.8	No	Yes
35.22	35.23	29.54	0.7±0.3	-111.3±4	-85.3±3	-80.3±3	85.5±2	-41.5±5	-12.0±2	44.0±4	18.9	No	Yes
34.96	34.99	30.05	0.8±0.1	-111.0±3	-86.2±4	-79.4±2	84.8±1	-40.8±4	-11.0±1	47.8±5	18.4	Yes	Yes
34.5	34.51	31.0	0.6±0.1	-106.8±8	-86.0±3	-79.9±2	84.6±1	-41.1±5	-11.3±2	46.8±5	19.8	Yes	Yes
34.13	34.14	31.74	0.6±0.2	-99.2±7	-84.9±4	-79.2±3	84.9±2	-40.1±4	-10.7±1	47.1±8	20.5	Yes	Yes
33.98	33.92	32.1	0.7±0.3	-100.7±10	-75.2±16	-66.2±12	83.3±2	-40.3±5	-9.9±1	57.7±6	17.9	Yes	Yes
33.3	33.24	32.97	0.6±0.4	-96.9±5	-74.1±15	-64.2±15	-83.7±1	-39.7±4	-10.0±2	58.1±4	19.0	Yes	Yes
32.97	33.03	34.0	1.7±0.6	-101.1±8	-55.0±8	-45.2±10	82.7±2	-36.4±6	-8.9±1	77.7±15	14	Yes	No
32.73	32.61	34.66	1.2±0.3	-93.3±6	-53.8±7	-44.6±7	82.6±2	-36.8±5	-9.0±2	61.1±6	20.0	Yes	Yes
31.97	32.01	36.02	1.5±0.2	-98.4±5	-47.8±4	-39.9±4	81.9±1	-35.6±6	-7.8±1	70.4±7	18.9	Yes	No
31.56	31.45	36.99	1.4±0.3	-92.4±7	-48.3±4	-41.8±4	81.3±2	-35.6±4	-8.4±2	68.6±10	20.7	Yes	Yes
30.97	31.02	38.01	1.5±0.4	-97.1±4	-47.4±3	-42.2±3	81.1±1	-36.1±4	-7.5±1	75.9±5	19.8	Yes	No
30.03	30.05	39.93	1.4±0.3	-90.0±12	-45.5±4	-41.4±4	81.2±2	-35.0±4	-6.7±1	87.5±8	18.6	Yes	No
29.02	28.99	41.99	1.4±0.4	-93.2±3	-45.8±3	-42.0±4	80.4±2	-35.2±4	-4.8±1	94.7±6	19.0	Yes	No
27.97	28.02	44.01	1.3±0.3	-97.0±4	-47.0±4	-41.5±3	80.6±2	-33.9±5	-2.9±3	107.8±5	17.3	Yes	No
26.97	27.03	45.99	1.4±0.3	-97.9±3	-47.5±3	-42.2±2	78.5±1	-34.1±5	-5.0±4	114.1±4	18.0	Yes	No

Table S7: Sucrose:Lysozyme 1:3

Suc [wt%]	Lys [wt%]	H_2O [wt%]	ΔC_p [J/g/K]	T_g^{onset} [°C]	T_g^{infl} [°C]	T_g^{end} [°C]	T_{den} [°C]	T_m^{on} [°C]	T_m [°C]	ΔH_m [J/g]	Am. H_2O [wt%]	Cryst.	Cold cryst.
17.52	52.45	30.04	1.0±0.4	-93.7±7	-54.51±4	-42.3±4	81.9±2	-37.2±4	-6.5±2	39.2±15	20.7	Yes	No
17.07	51.19	31.74	1.4±0.5	-103.7±10	-55.19±3	-41.6±4	79.0±2	-37.2±3	-4.2±1	52.4±19	19.0	Yes	No
16.56	49.59	33.85	1.0±0.3	-96.6±9	-56.7±4	-42.5±3	78.9±2	-36.8±4	-4.8±1	56.9±8	20.3	Yes	No
16.28	48.83	34.9	1.7±0.7	-103.9±8	-57.55±3	-42.5±3	76.2±3	-36.8±3	-3.0±2	69.7±8	17.7	Yes	No
16.06	48.13	35.81	0.8±0.2	-86.4±10	-56.05±3	-42.4±3	76.2±1	-37.8±4	-4.0±1	70.5±7	18.6	Yes	No
15.68	47.05	37.27	1.1±0.7	-92.1±22	-57.14±5	-42.2±5	75.4±2	-37.8±5	-0.3±4	78.9±10	17.9	Yes	No
15.54	46.53	37.93	0.8±0.4	-91.5±11	-57.09±4	-45.9±4	76.2±2	-37.2±7	-4.8±3	70.2±5	21.4	Yes	No
15.06	45.16	39.78	1.0±0.4	-87.9±12	-58.08±4	-42.3±3	74.2±2	-38.2±6	-3.1±1	82.3±4	20.1	Yes	No
14.5	43.43	42.07	0.9±0.4	-91.7±10	-61.02±4	-46.2±4	74.2±2	-37.7±6	-3.8±3	86.0±4	22.0	Yes	No
14.05	42.1	43.86	0.7±0.3	-85.6±10	-57.83±3	-41.9±2	73.1±1	-34.3±6	-0.6±2	85.2±16	24.6	Yes	No
13.52	40.54	45.94	0.7±0.2	-84.4±7	-60.31±3	-41.6±3	73.1±2	-34.3±5	-1.5±2	98.8±7	23.2	Yes	No

3 Additional plots glass transition temperature

To perform an extensive analysis to compare the stabilizing effect of sucrose and trehalose, both at lower as well as higher temperatures, the data was plotted both as a function of the protein and sugar content, respectively. Figures S5 and S6 display the glass transition temperature, T_g, as a function of the protein content (wt%) for the sucrose and trehalose containing samples, respectively. The black diamonds, in Figure S5, correspond to a sugar:protein weight ratio of 1:3, where the T_g is seen to decrease with increasing water content. The blue squares in Figures S5 and S6 represent the samples with a sugar:protein weight ratio of 1:1, which show a T_g slightly higher for the trehalose-containing samples. However, it should be mentioned that the homogeneity of the trehalose samples was more difficult to ensure due to a slightly more limited solubility. The red circles correspond to a sugar:protein weight ratio of 3:1. Comparing T_g of the two disaccharide systems it can be concluded that there is little to no difference between the two systems.

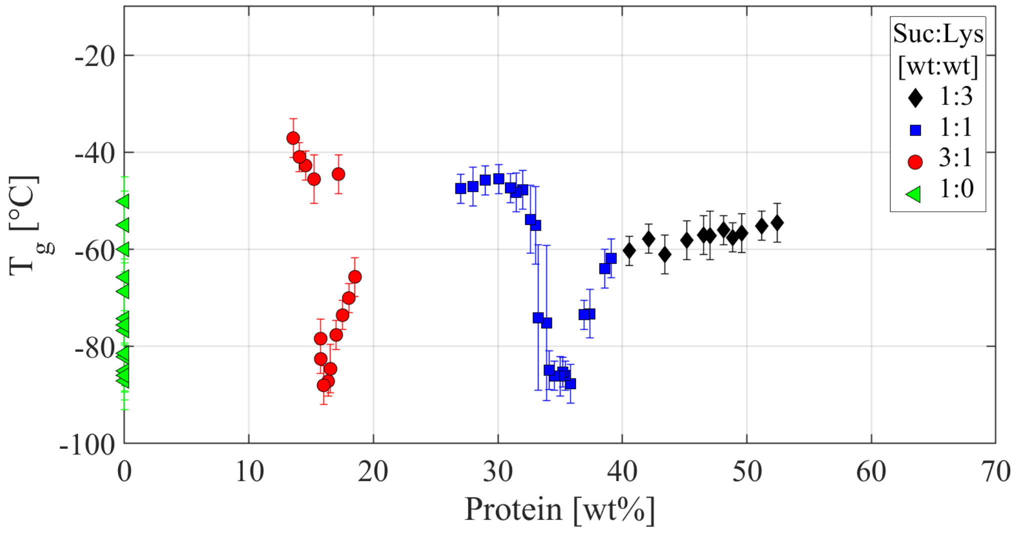

Figure S5: The T_g as a function of the protein content (wt%). The black diamonds, blue squares, red circles, and green triangles represent sucrose:lysozyme weight ratios 1:3, 1:1, 3:1, and 1:0, respectively.

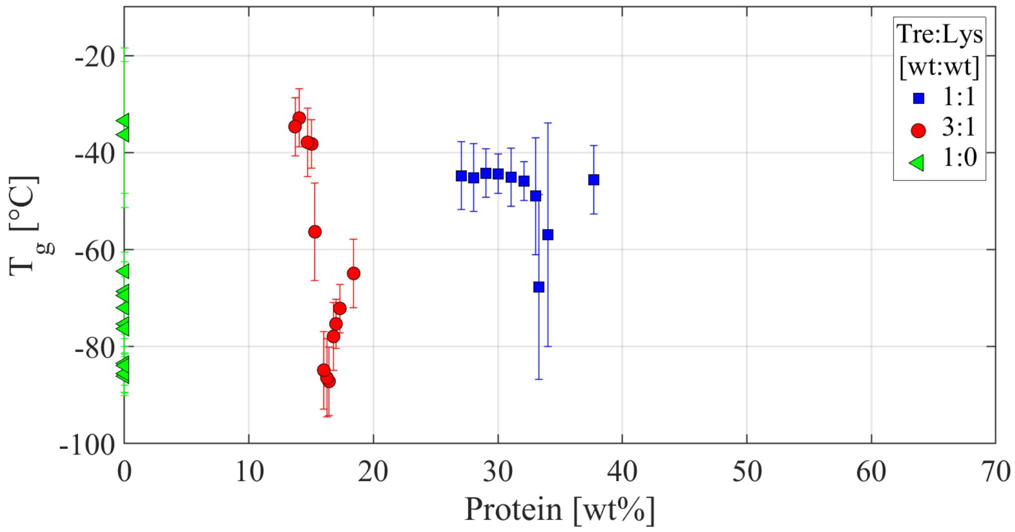

Figure S6: The T_g as a function of the protein content (wt%). The blue squares, red circles, and green triangles represent trehalose:lysozyme weight ratios 1:1, 3:1, and 1:0, respectively.

Figures S7 and S8 present the T_g as a function of the sugar content (wt%). For the sugar:protein weight ratios 3:1 and 1:0 (and also 1:1 in the case of sucrose) clear "V-shaped" curves are visible for both disaccharides due to that the left branch of the "V" corresponds to samples that crystallized during cooling, which explains the abrupt increase of T_g.

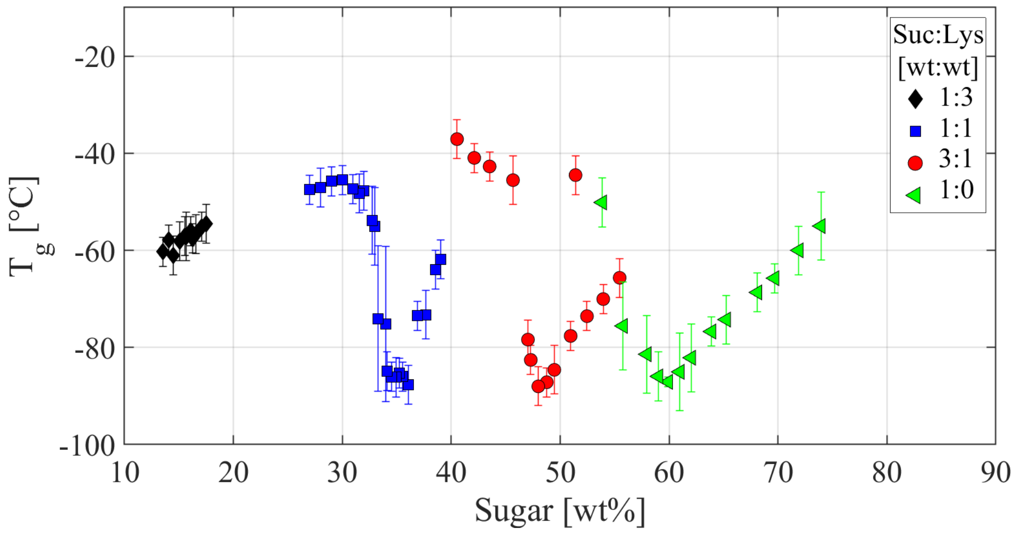

Figure S7: The T_g as a function of the sugar content (wt%). The black diamonds, blue squares, red circles, and green triangles represent sucrose:lysozyme weight ratios 1:3, 1:1, 3:1, and 1:0, respectively.

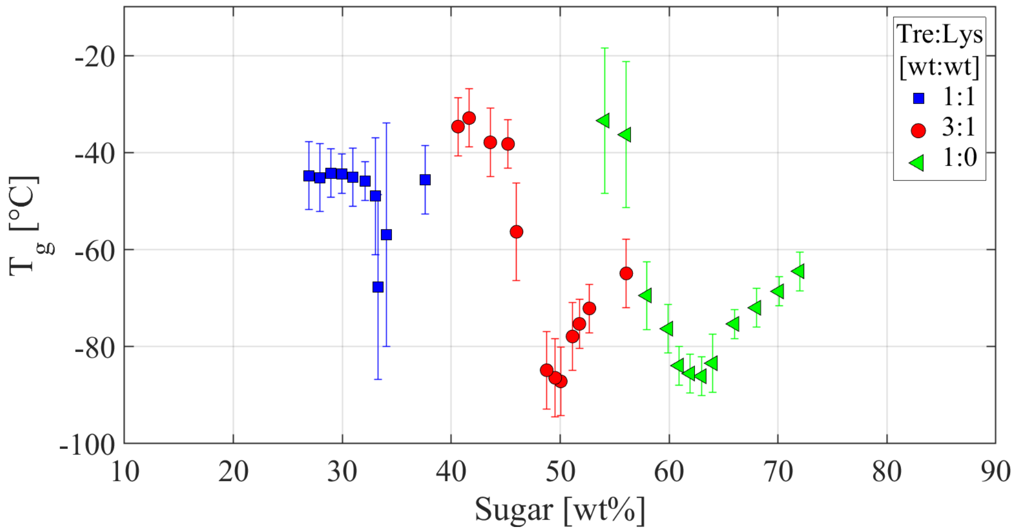

Figure S8: The T_g as a function of the sugar content (wt%). The blue squares, red circles, and green triangles represent trehalose:lysozyme weight ratios 1:1, 3:1, and 1:0, respectively.

4 Additional plots denaturation temperature

Figures S9 and S10 show the T_{den} of the protein lysozyme as a function of the protein content (wt%). It can clearly be seen that the T_{den} increases with decreasing water content for all different sugar:protein ratios, although this trend is strongest for the sucrose containing samples. Furthermore, it is possible to observe that the T_{den} strongly depends on the sugar:protein ratio.

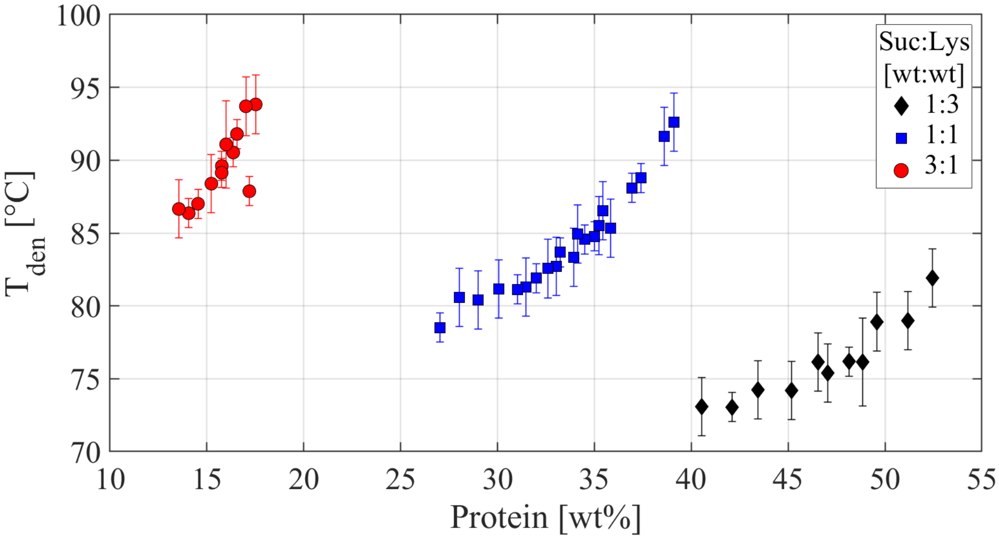

Figure S9: The T_{den} as a function of the protein content (wt%). The black diamonds, blue squares, and red circles represent sucrose:lysozyme weight ratios 1:3, 1:1, and 3:1, respectively.

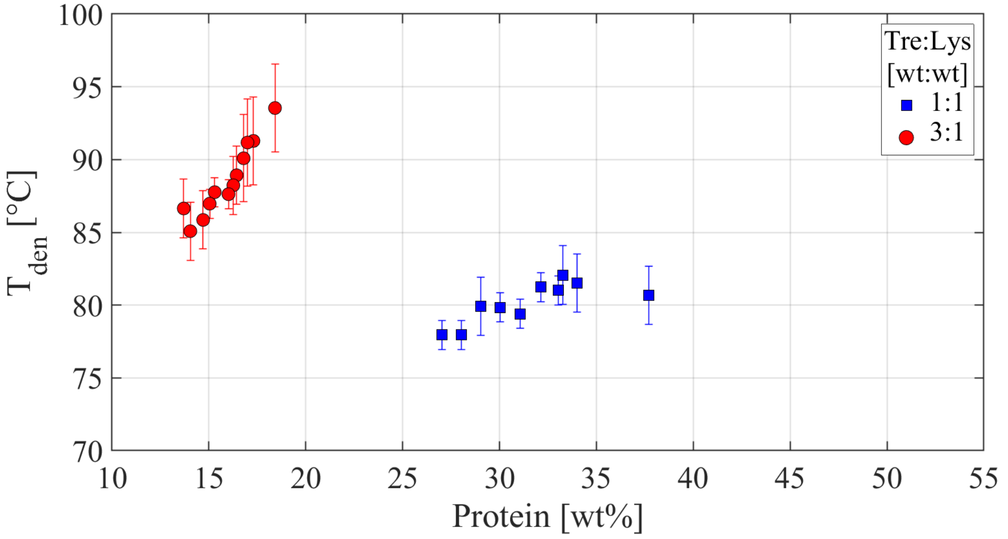

Figure S10: The T_{den} as a function of the protein content (wt%). The blue squares, and red circles represent trehalose:lysozyme weight ratios 1:1, and 3:1, respectively.

In Figures S11 and S12 the T_{den} is displayed as a function of the sugar content (wt%). Here it is obvious that the T_{den} increases with increasing sugar content and also with increasing sugar:protein ratio. This is reasonable since more sugar molecules per protein molecule can assist in maintaining preferential hydration of the protein.

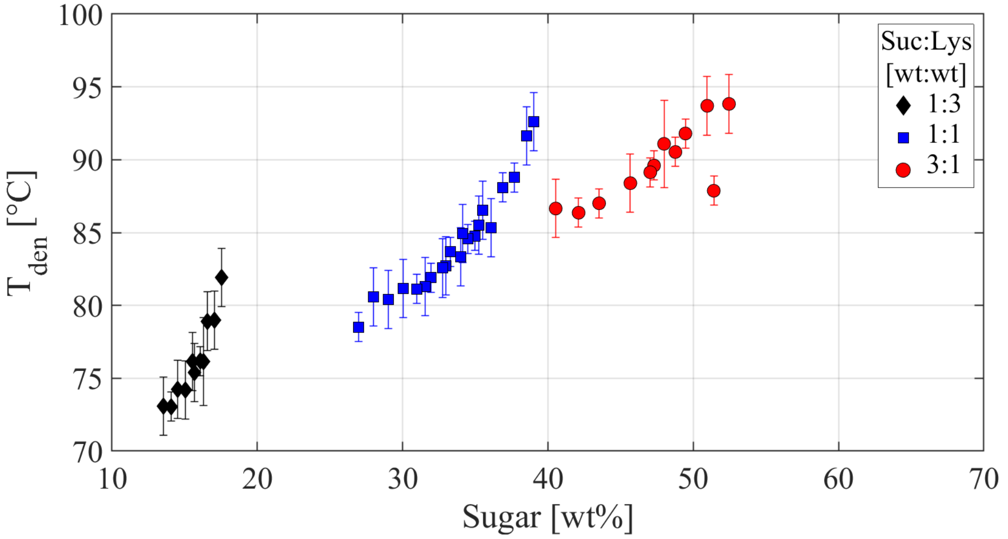

Figure S11: T_{den} as a function of the sugar wt%. The black diamonds, blue squares, and red circles represent sucrose:lysozyme weight ratios 1:3, 1:1, and 3:1, respectively.

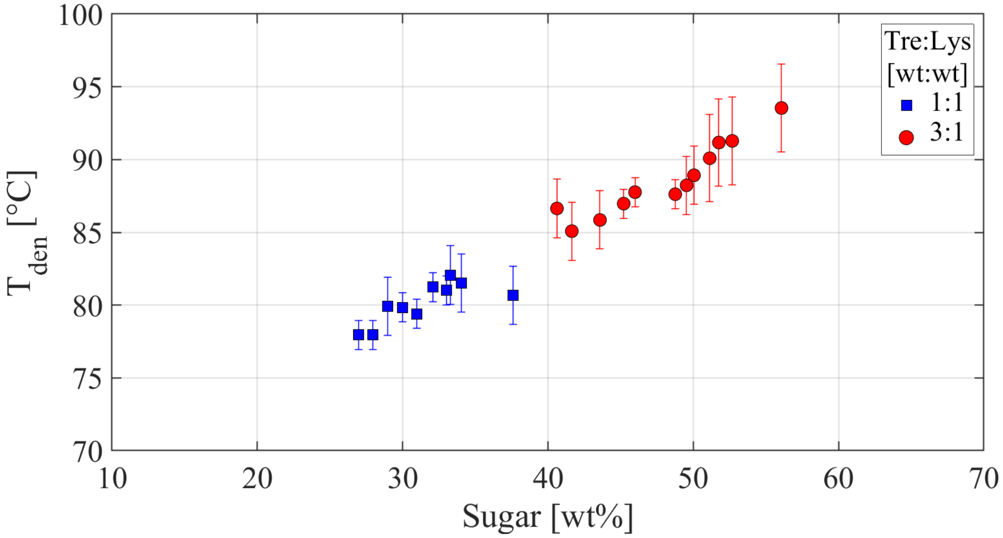

Figure S12: T_{den} as a function of the sugar wt%. The blue squares, and red circles represent trehalose:lysozyme weight ratios 1:1, and 3:1, respectively.
